# The effects of Core Stability Exercises and Mulligan’s mobilization with movement techniques on sacroiliac joint dysfunction

**DOI:** 10.3389/fphys.2024.1337754

**Published:** 2024-04-18

**Authors:** Huiqian Yan, Peng Zhao, Xuanhui Guo, Xiao Zhou

**Affiliations:** ^1^ Sports Rehabilitation Research Center, China Institute of Sport Science, Beijing, China; ^2^ College of Sports Medicine and Physical Therapy, Beijing Sport University, Beijing, China

**Keywords:** core stability, exercise therapy, musculoskeletal manipulations, sacroiliac joint, low back pain

## Abstract

**Purpose:**

Sacroiliac joint dysfunction (SIJD), while being the primary contributor to low back pain, is still disregarded and treated as low back pain. Mulligan’s Mobilization with Movement (MWM) Techniques and Core Stability Exercises (CSE) are often used to treat low back pain. There is not much evidence that it is effective in SIJD. To evaluate the effectiveness of CSE coupled with MWM (CSE + MWM) in the treatment of SIJD.

**Methods:**

39 patients with SIJD were recruited and randomly divided into distinct groups as follows: control group (n = 13), CSE group (n = 13) and CSE + MWM group (n = 13). The Numerical Pain Rating Scale (NPRS), the Roland Morris Disability Questionnaire (RMDQ), the Range of Motion (ROM), the Pressure Pain Threshold (PPT) and the pelvic tilt angle asymmetry ratio in the sagittal plane (PTAR) were used to gauge the intervention’s success both before (M0) and after (M1) it. All experimental data were statistically analyzed.

**Results:**

The SIJ-related pain metric significantly decreased in both the CSE + MWM group and the CSE group between M0 and M1, as determined by the NPRS and RMDQ. Between M0 and M1, The CSE group’s left axial rotation ROM and lumbar flexion ROM were significantly decreased. The CSE + MWM group’s extension ROM and left lateral flexion ROM both significantly increased between M0 and M1. In the difference variable (M1-M0), the CSE + MWM group substantially outperformed control group in the left lateral flexion ROM and outperformed the CSE group in the left axial rotation ROM.

**Conclusion:**

In individuals with SIJD, CSE + MWM is beneficial in lowering pain, disability, and function. Treatment with CSE and MWM approaches for SIJ appears to boost this efficacy.

## 1 Introduction

According to the number of years people live with a handicap, disability is the main cause of low back pain worldwide (Vos et al., 2012). It is estimated that between 70% and 85% of people in the west may experience low back pain at some time in their lives each year (Andersson, 1999). Low back pain has been linked to sacroiliac joint dysfunction (SIJD), which is frequently discussed (Alderink, 1991). A number of clinical disorders, aberrant joint mobility, and misalignment of the joint can all lead to dysfunction (Gartenberg et al., 2021). And pain in the low back resulting from a change in the typical joint motion attributed to either hypermobility or hypomobility is known as SIJD (Cohen and Rainville, 2002). According to published data, 15%–25% of patients with nonspecific low back pain have SIJD (clinical examination, screening methods, and intraarticular test blockages) (Cohen, 2005; Thawrani et al., 2019). Consequently, Clinical consideration must be given to primary low back pain caused by SIJD. Despite the fact that SIJD significantly increases the risk of chronic low back pain, it is commonly misdiagnosed or treated inadequately. In addition to being disabling for the person who has it, SIJD costs society money since it keeps sufferers from working and has a detrimental effect on their families (Dutta et al., 2018).

Relieving discomfort and addressing the underlying dysfunction are the goals of treatment for SIJD (Cohen et al., 2013; Peebles and Jonas, 2017). Additionally, there are various intervention techniques used in the physical therapy for SIJD. The use of manual therapy, patient education, exercise therapy, taping, and electrotherapeutic modalities are a few examples of such interventions (Al-Subahi et al., 2017; Gartenberg et al., 2021). The primary goals of physical therapy are lumbar stabilization, SIJ asymmetry repair, and muscular imbalance management (Zelle et al., 2005).

Few studies have examined the efficacy of exercise therapy in SIJD, despite the fact that it is recommended as an effective way for treating low back pain (Delitto et al., 2012; Maher et al., 2017; Van Wambeke et al., 2017). Core Stability Exercises (CSE) are a common form of exercise therapy used to treat low back pain. CSE can reduce disability, relieve pain, improve muscular function, support lumbar stability, improve mental health, and lower the risk of recurrent injury (Owen et al., 2019; Kreiner et al., 2020).

Additionally, manual therapy successful complementary and alternative medicine treatment for those with low back pain (Waqqar et al., 2016; García-Pealver et al., 2020). A joint’s soft tissue can be stretched during manual treatment, which can also increase Range of Motion (ROM), lessen swelling around the joint and muscle spasms, rectify joint flaws, and manage pain (Orakifar et al., 2012; Nejati et al., 2016). For musculoskeletal problems including low back pain and other illnesses, Mulligan approaches, such as the Sustained Natural Apophyseal Glide (SNAG) and Mobilization with Movement (MWM), are used routinely in physical therapy practice (Pourahmadi et al., 2018; Stathopoulos et al., 2019). MWM is a manual treatment technique created by Brian Mulligan that treats joint positioning defects by combining accessory mobilizations with natural motion (Mulligan, 2004).

An quick reduction or cessation of pain and an increase in ROM are two of the fundamental principles of therapeutic care outlined by the MWM (Mulligan, 2004). Brian Mulligan uses the idea of positional defects to explain how pain is reduced. Mulligan claims that minor postural errors are brought on by injury and muscular imbalance (Mulligan, 2004). Combining this joint gliding with a natural spinal motion (lumbar flexion, for example) and advocating for resolving (biomechanical) joint issues that could result in symptoms (Exelby, 2002; Mulligan, 2004). However, just one study—that of Krzyzanowicz et al. (2015) had thus far looked into how a SIJD sample’s pain and disability are affected by MWM approaches and selective functional mobility assessment, including CSE. While this study lacks functional implications. Therefore, it is clear that more research is necessary to determine whether function is changed by using the MWM on the SIJ.

Despite the significant occurrence of SIJD, there are no recommendations or effective treatments for this syndrome yet. Previous research has mostly been on reports of CSE used in conjunction with Mulligan techniques to treat low back pain (rarely including SIJD) (Hussien et al., 2017; Pourahmadi et al., 2018; Bhat et al., 2021). This inspired us to research the impact of CSE alone and combined with MWM (CSE + MWM) on pain, disability, and function in SIJD patients.

## 2 Materials and methods

### 2.1 Sample size calculation

The current investigation was conducted after receiving approval from the Institutional Ethical Committee. The number of participants needed in each group for an 80 percent power and a = 0.05 significance level was calculated using the G-Power 3.1.9.2 program. According to the findings of the study that was referred to (Javadov et al., 2021), each group needed to have a minimum of 10 participants and an effect size of 0.62.

### 2.2 Participants

A single-blind, randomized, and controlled study was intended. On 28 May 2021, Sports Science Experiment Ethics Committee of Beijing Sport University granted its clearance (ID: 2021085H). The participants signed the “Informed Consent” in accordance with the Helsinki Declaration after receiving the sample and information selection. The study was conducted from March 2021 to June 2021.39 participants (2 men and 11 women in each group) in all were chosen from the Beijing Sport University and added after meeting the study’s inclusion requirements. To participate in the study, participants signed an informed permission form, after which they were divided into three groups at random. A randomization table produced by a web-based computer software was used for the randomization. In a 1:1 ratio, the participants were split between the control group, CSE group, and CSE + MWM group.

The IASP’s (International Association for the Study of Pain) suggested diagnostic standards were used to make the diagnosis: patients experiencing SIJ pain, which is pain in the hips, groin, or possibly the lower extremities; SIJ-specific discomfort that reacted to particular provocation tests (Merskey and Bogduk, 1994). Inclusion criteria were age between 18 and 60 years old, no fractures or surgery in the lumbosacral or pelvic region in the prior to the study, no pregnancy and lactation, no radicular pain or radiculopathy, no spinal pathology, no osteoporosis, no physical therapy for 3 months prior, non-injection of corticosteroids or anesthetics in the SIJ during the previous month, non-injection of corticosteroids or anesthetics in the SIJ in the preceding month, lack of sacroiliac infection or sacroiliitis, and testing positive results in three or more of the following (Levangie, 1999).

a. Faber test
b. Distraction test
c. Thigh thrust test
d. Gaenslen test
e. Sacral thrust test

Participants were excluded if they made their pain worse, used alternative painkillers, or stopped the intervention program for any reason. The study protocol is given in Supplementary Figure S1.

### 2.3 Intervention

Over the course of 6 weeks, all participants in the control group, the CSE group, and the CSE + MWM group attended a total of 18 intervention sessions. All interventions were administered by three certified therapists with a Master’s degree in Physiotherapy who received unified training from sports medicine specialists prior to the start of the study.

#### 2.3.1 Core Stability Exercises

The identical stabilization program-based exercises were given to all trial participants. For a 6-week period, participants were expected to attend three times per week of supervised therapy sessions. Each session lasted between thirty and 45 minutes. Each participant began the workout with a 10- to 15-min warm-up that included stretching exercises and stationary cycling. Participants in both the MWM group and the CSE + MWM group got a 6-week intervention as well as CSE ball instruction, with a focus on strengthening the deep abdominal muscles and gluteus maximus muscle. The CSE program included five movements (the abdominal drawing-in maneuver, quadruped arm and lower extremity lift, side-bridge, bridge and double knee flexion, unilateral-bridge) (Ekstrom et al., 2007; Nejati et al., 2019), and the therapist gave the full routine based on the prescribed guidelines. There were two phases to these exercises. Every 3 weeks, a new phase began. During this intervention time, there were two phases: the first involved learning the abdominal drawing-in maneuver, the quadruped arm and lower extremity lift, and the unilateral bridge; the second involved learning five exercises during the following 3 weeks. Patients in each group were instructed to keep their current lifestyles during the intervention period without participating in any rehabilitation programs.

#### 2.3.2 Mulligan’s mobilization with movement techniques

Joint mobilization was accomplished using the posterior innominate and anterior innominate techniques (Mobilization with Movement). Based on the physical examination (such as palpation, dynamic testing, and pain reaction), the posterior and anterior innominate of the MWM were determined (Krzyzanowicz et al., 2015). The same therapist applied a Mulligan MWM based on the fundamental ideas Mulligan outlines in his text to each participant (Mulligan, 2004). Based on the findings of the physical examination, each participant was assigned either an anterior or posterior rotation of the ilium (Mulligan, 2004). An anterior MWM was used to address the anterior rotation of the ilium. The patient was asked to complete a prone press-up while the MWM was being conducted by grasping and rotating the affected ilium posteriorly while the sacrum was stabilized. If there is no pain, have the patient extend passively while lying down. A posterior MWM was used to correct the ilium’s posterior rotation. In order to prevent the patient from rolling, the therapist applied counterpressure to the other ilium while mobilizing the SIJ anterolaterally through the thenar eminence on the posterior superior iliac spine. During each treatment session, the MWM was applied without weight bearing for three sets of 10 repetitions, Mulligan has previously stated that MWM should not cause any discomfort (Mulligan, 2004). Under the guidance of a skilled sports medicine specialist, the therapist performs these techniques during the initial session.

### 2.4 Outcomes

The three groups were assessed twice during the intervention: at the beginning (M0, prior to the intervention) and at the end (M1, following the intervention). Participants were asked to fill out a questionnaire at the outset with demographic and descriptive data, including their age, sex, height, weight, and Body Mass Index (BMI).

#### 2.4.1 Pain

The Numerical Pain Rating Scale (NPRS) is utilized, pain was evaluated. The method that is most frequently used to gauge pain is the NPRS. By marking a spot on a 10 cm horizontal line, the intensity of the pain was calculated. The line’s left side (0 points) was meant to represent no pain, while its right side (10 points) was meant to represent the most extreme pain (Von Korff et al., 2000).

#### 2.4.2 Disability

To determine the level of functional disability, the Roland Morris Disability Questionnaire (RMDQ), which comprises 24 items, was employed. These questions evaluated one’s level of physical fitness and activity, sleep and rest, social psychology, housekeeping, eating habits, and pain frequency. Every item has a number ranging from 0 (No) to 1 (Yes). The degree of disability rises as the overall score does (Roland and Morris, 1983).

#### 2.4.3 Function

The active Range of Motion (ROM) of the lumbar spine was evaluated using the wireless microFET6 Dual Digital Inclinometer (Microfet6, Hoggan Health Industries Inc., West Jordan, UT) (Saur et al., 1996). An inclinometer is positioned at T12 and another is positioned at S1. It is possible to isolate lumbar ROM while implicitly eliminating hip joint and sacral ROM. The Dual Digital Inclinometer measurement approach was used to the inferior S1 and superior T12 spinous processes (Tousignant et al., 2002). The patient was told to do active lumbar movements while keeping their knees straight.

The Pressure Pain Threshold (PPT) was measured using a hand-held algometer (Pain Diagnostic Treatment, New York, USA) with a rubber tip that was 1 cm^2^ broad and compressed the region (Orakifar et al., 2012). Since SIJD frequently affects the posterior superior iliac spine (PSIS), we decided to evaluate PPT values there (Triano et al., 1997). After the participants verbally reported pain, measurements were collected by positioning the algometer’s tip perpendicular to the PSIS and applying posterior to anterior pressure at a rate of 1 kg/cm^2^ per second (Shearar et al., 2005). When the participants felt discomfort, they were instructed to respond with a “now”. The PPT number is the maximum pressure that was applied, and the researcher stopped immediately. Higher readings suggested either a higher pain threshold or less sensitivity to pain (Orakifar et al., 2012).

The pelvic tilt angle asymmetry ratio in the sagittal plane (PTAR) was calculated by the left/right anterior pelvic tilt angle, whose evaluation has shown a good inter-rater correlation coefficient (ICC = 0.76–0.77) and intra-rater correlation coefficient (ICC = 0.78–0.83) (Yu et al., 2020). Using the PA200LE Station Posture Assessment. System (Big Sports, Japan), participants’ standing postures’ anterior pelvic tilt angles (angle between the anterior superior iliac spine and the posterior superior iliac spine) were measured (Yu et al., 2020; Li et al., 2022). The participants were instructed to stand barefoot naturally in their underwear for the measurements, and the device recorded their posture. The median line allows the apparatus to discriminate between the participant’s left and right side postures. The anterior pelvic tilt angle was measured and averaged for the left and right sides in this investigation. Similar to the methods used by Yu et al. (2020), to determine the relative ratio between the two sides, the pelvic asymmetry ratios of each anterior pelvic tilt angle were first determined by dividing the left side’s parameter by the right side’s parameter. After then, 1 was deducted in order to equalize the ratio. The following formula was utilized to calculate pelvic asymmetry:

$$PTAR\;\left(\%\right)=\left|\;\left(\frac{left\;anterior\;pelvic\;tilt\;angle}{right\;anterior\;pelvic\;tilt\;angle}\right)-1\;\right|\times 100$$

### 2.5 Blinding

The therapist in charge of providing manual treatment was not blinded to randomization because of the nature of this research. After participants assignment to each group, the exercise was carried out by three therapists. Assessors, participants, and the person assigning them will all be blind to the therapy groups, though. Conditions for blindness should only be activated in the event of a medical emergency. Before and after the intervention phase, participants were assessed by a blind assessor.

### 2.6 Statistical analysis

Using the software IBM SPSS Statistics^®^ version 26.0, descriptive and inferential statistical analyses were carried out. Before conducting a one-way analysis of variance, the Shapiro-Wilk and Levene tests were employed to evaluate the distribution and homogeneity of variance (ANOVA). One-way Given that the study’s variables had a normal distribution, an analysis of variance (ANOVA) was used to compare the groups in the quantitative variables. A paired-sample *t*-test was used for an intragroup comparison test. The α level was set at *p <* 0.05.

## 3 Results

36 patients were submitted to the final assessment out of the 39 individuals who matched the inclusion criteria. The control group (n = 13), the CSE group (n = 11), and the CSE + MWM group (n = 12) were formed from these patients. Due to their irregular attendance and failure to finish the exercise regimen by week six, 2 CSE group participants were specifically excluded. In addition, 1 CSE + MWM group participant was excluded due to their occupation. A total of 36 participants from each group were included in the analysis.

When analyzing the participant characteristics with relation to demographic (age) and anthropometric (weight, height, and BMI) data in Supplementary Table S1, there were no statistically significant differences between the groups (*p* > 0.05). Supplementary Table S2 lists the Mean SD of the M0 and M1 NPRS, RMDQ, flexion ROM, extension ROM, right lateral flexion ROM, left lateral flexion ROM, right axial rotation ROM, PPT and PTAR.

The VAS in M0 did not significantly differ between the groups. Between moments (from M0 to M1), VAS significantly decreased in the CSE group and CSE + MWM group (*p* < 0.05), but not in the control group (*p* = 0.053). In the difference variable, there were no groups (*p* = 0.255) that differed significantly (M1-M0).

The RMDQ in M0 did not significantly differ across the groups. Between moments (from M0 to M1), RMDQ significantly decreased in the CSE group and the CSE + MWM group (*p* < 0.05), but not in the control group (*p* = 0.421). Between the groups, there were no discernible differences (*p* = 0.056) in the difference variable (M1-M0).

The flexion ROM and extension ROM in M0 did not significantly differ across the groups. Between moments (from M0 to M1), there was a significant increase in extension ROM in the CSE + MWM group (*p* = 0.032) and a significant decrease in flexion ROM in the CSE group (*p* = 0.041), whereas in the control group there were no significant differences (*p* > 0.05). In the difference variable, there were no groups (*p* > 0.05) that differed significantly (M1-M0).

The right lateral flexion ROM and left lateral flexion ROM in M0 did not significantly differ across the groups. Left lateral flexion ROM increased significantly in the CSE + MWM group (*p* = 0.007), but not in the control group or the CSE group (*p* > 0.05). Left lateral flexion ROM was significantly different between the groups (*p* = 0.008) in the difference variable (M1-M0). When compared to the control group, the CSE + MWM group’s left lateral flexion ROM was considerably higher (*p* = 0.002) than that of the control group (M1-M0).

The right axial rotation ROM and left axial rotation ROM in M0 did not significantly differ across the groups. While there was no significant difference between the left axial rotation ROM in the control group and the CSE + MWM group (*p* > 0.05), there was a significant decrease in the left axial rotation ROM in the CSE group (*p* = 0.006) between moments (from M0 to M1). In the difference variable, there were no groups (*p* > 0.05) that differed significantly (M1-M0). However, when contrasting the CSE + MWM group with the CSE group, the CSE + MWM group (*p* = 0.029) considerably outperformed the CSE group in the difference variable for left axial rotation ROM (M1-M0).

The PPT in M0 did not significantly differ across the groups. Between moments (from M0 to M1), PPT significantly increased in the control group (*p* = 0.038), but not in the CSE group and CSE + MWM group (*p* > 0.05). In the difference variable, there were no groups (*p* = 0.560) that differed significantly (M1-M0).

The PTAR in M0 did not significantly differ across the groups. Between moments (from M0 to M1), there were no significant difference in PTAR among the three groups (*p* > 0.05). In the difference variable, there were no groups (*p* = 0.511) that differed significantly (M1-M0).

## 4 Discussion

In order to examine how CSE and CSE + MWM affected pain, disability, and function in SIJD patients, this study compared their effects. We believe that this study is one of the first to compare various SIJD treatment options available in China. Both the CSE group and the CSE + MWM group had improvements in outcomes of NPRS, RMDQ, and ROM in the short term, although there were no statistically significant differences between the groups for the outcomes NPRS and RMDQ (*p* > 0.05).

On the NPRS, both pain and disability showed a clinically significant improvement in the CSE group and the CSE + MWM group. The therapeutic use of the MWM techniques’ mechanism is still not completely understood (Buran Çirak et al., 2021). There are clinical and biomechanical laboratory investigations in the literature. It has been found that correcting intra-articular position abnormalities and neurophysiological mechanisms can significantly lessen pain. Vicenzino et al. (2007) discovered that the mechanical basis of the most prevalent theory put forth for this rapid pain relief effect is based on the notion that bone alignment abnormalities exist and that MWM can fix these faults. When Krzyzanowicz et al. (2015) published a case study of 3 recreational dancers with SIJ discomfort, the results were similar to those of this study. The MWM was applied nonweight bearing for three sets of 10 repetitions during each treatment session. And they performed stability exercises based on selective functional movement assessment for 2-8 treatment sessions. Sustained and significant changes in outcomes of NPRS and Physically Active (DPA) scale scores occurred during the treatment phase of the trial when compared to baseline data.

MWM were clinically superior to improve pain and function, even though both groups in the current trial improved comparably over the short term. However, there were no appreciable variations in the groups’ NPRS and RMDQ results. Hussein et al. (2017) evaluated the effects of adding SNAG to a standard therapy program for chronic nonspecific low back pain, which is at odds with this study. Both groups’ pain was reported to have decreased, however the researchers came to the conclusion that adding SNAG to traditional physiotherapy led to greater improvements in the Visual Analog Scale (VAS) and Oswestry Disability Index (ODI). However, in Javadov et al. (2021), lumbar exercises were given to Group 3, a home-based lumbar exercise program were combined with SIJ manipulation in Group 2, and SIJ manipulation and a home-based exercise regimen for the SIJ were employed in Group 1. On the twenty-eighth and 90th days, all three groups with SIJD had a significant decline in VAS and Modified ODI. On days fourteen, twenty-eight, and ninety, Group 2’s VAS was significantly lower than Group 3’s. However, when Modified ODI were evaluated, there were no appreciable changes between Groups 2 and 3 on the twenty-eighth and 90th days. These results are consistent with those of the current investigation. The shorter length of therapy used in our investigation may be the reason why the findings differ from Javadov’s study.

In our study, the CSE combined with MWM was not more effective at treating SIJD than CSE and control. The difference variable of the three groups improve the value is low, there is no significant difference between the groups. This finding may be related to lower participant scores on the baseline NPRS and RMDQ. Additionally, a tiny change or improvement in the statistical model used to calculate random grouping is regarded as negligible. It was previously discussed how CSE + MWM might not have been any more effective in week 6.

In our investigation, only the CSE group showed a reduction in left axial rotation ROM and lumbar flexion ROM. The objective of CSE is the activation of deep trunk muscles, in order to restore the control and coordination of these muscles (O'Sullivan et al., 1997). Deep trunk muscles are pre-activated throughout these exercises, which advance to more difficult and practical activities that combine the activation of both deep and global trunk muscles (O'Sullivan et al., 1997). Stability is increased when the core muscles are stimulated to become more rigid (Brumitt et al., 2013). According to Shamsi et al. (2017), spinal loads and spinal stability will both rise if muscular activity levels rise to a significant degree. An antagonist muscle’s coactivity, for example, enhances spinal stability; nevertheless, excessive coactivity (together with spinal stability) raises spinal loads and stiffness, which can interfere with agile movements. In addition, these results in our study are consistent with the results of previous studies that showed a decrease in joint motion in some directions after 6 weeks of core stability home training in patients with joint limitation (Moreside and McGill, 2012). The CSE used in our study is similar to the core stability home training used in this study by Moreside and McGill (2012), which may be the reason for the decline in ROM. Therefore, we infer that the degree of spinal stability that CSE brings to the spine may affect spinal activity, and more research is needed to explore the effect of CSE on the degree of spinal stability in the future.

Only the CSE + MWM group in our study experienced an increase in lumbar extension ROM and left lateral flexion ROM. There are several potential reasons why the ROM increased after MWM was applied. Participants in the Mulligan techniques assume a non-weight-bearing stance. This makes the patients think that the resting position is a kind of therapy that might make their pain go away during later activities. As a result, it is possible to assume that the MWM application caused the increase in extension ROM because MWM was used in the repetitive passive extension action (Konstantinou et al., 2007). Mulligan approach, which is suggested to move an articular surface to its matching surface during motion, is another theory that might apply. Positional errors are thought to be the main source of discomfort, and if they are corrected, the pain and muscle spasms around the injured joints should subside (Vicenzino et al., 2007). Trunk muscle recruitment, posture, movement pattern, and respiration were all assessed and adjusted during this procedure (Saragiotto et al., 2016).

Our study found that when compared to control, CSE + MWM had a more advantageous impact on enhancing left lateral flexion ROM and left axial rotation ROM than CSE alone. Regarding the improvement of joint ROM in particular peripheral joint pathologies, a systematic review and meta-analysis conducted by Stathopoulos et al. (2019) revealed findings that are similar to those of this study, concluding that peripheral joint MWM appears to produce better therapeutic results in comparison to sham, passive, other active, or no therapeutic approach. In order to evaluate the information regarding the effectiveness of Mulligan techniques for treating low back pain, Pourahmadi et al. (2018) did a systematic review and meta-analysis. In this study, individuals with low back pain may have less pain and impairment thanks to the Mulligan techniques of manual therapy. However, the study cited above looked at Mulligan’s influence on a group of people who had symptoms of low back pain. Our study focuses primarily on an often mentioned cause of low back pain, offering proof of changes in mechanical parameters following the use of manual therapy.

Additionally, our study demonstrated that there is no discernible change in flexion ROM and extension ROM among groups. Moutzouri et al. (2008) conducted a double-blinded investigation with 49 asymptomatic volunteers that had findings that were comparable to those of our study. Randomly chosen subjects were given either SNAG mobilization or a phony mobilization. When lumbar flexion ROM was compared between each of these groups, no discernible differences were found. This discovery, however, went against the conclusion that SNAG approaches are superior than Mechanize in improving lumbar ROM when treating chronic mechanical low back pain (Waqqar et al., 2016). A further study by Bhat et al. (2021), randomly allocated 65 patients with subacute or chronic non-specific low back pain to receive strengthening exercises plus either Myofascial release therapy or SNAG for six therapy sessions spaced over a week. As a result, for limited lumbar flexion ROM, SNAG perform better than Myofascial release therapy in the short term. We think that this is the cause of the unnoticed improvement in flexion ROM and extension ROM, given these findings demonstrated SNAG treatment had an immediate and short term effect on lumbar ROM. However, our intervention lasted for 6 weeks rather than just one.

The PPT of CSE group and CSE + MWM group was not significant within or between groups in our results. Nonetheless, a study had demonstrated that comparable methods effectively reduce pain in areas of the spinal column (Sipko et al., 2018). And 129 nursing assistants participated in a trial conducted by Moreira et al. (2021). A reference group and an intervention were randomized to 90 participants. The 12-week intervention consisted primarily of spine stabilization exercises. Consequently, an intervention group exhibited a considerable rise in PPT in the low back as compared to a reference group. Furthermore, PPT values rose dramatically in the spine from the cervical to the lumbar regions, according to Vanderweeën et al. (1996). The lumbar and SIJs have a lower density of mechanoreceptors and nociceptors than the higher regions of the spine (Vanderweeën et al., 1996; Orakifar et al., 2012). This could account for the current study’s nonsignificant findings. And the current study’s findings are consistent with those published by Shearar et al. (2005), who discovered that mobilization and manipulation seemed to raise the PPT in the SIJ.

In this study, the PTAR of CSE group and CSE + MWM group was insignificant within or between groups. Barbosa et al. (2013), on the other hand, found that 7 participants with low back pain and pelvic anteversion underwent an 8-week treatment. They consented to the manipulation of the SIJ and then contracted their hamstrings and quadriceps eccentrically and concentrically. Consequently, the pelvic angles indicated a noteworthy reduction in size between the evaluations. A three-paradigm intervention model was described by Cottingham and Maitland. (1997) as one that might be modified for the treatment of persistent idiopathic low back pain. Manual methods and physical activities to rectify particular biomechanical alignment errors (such as pelvic asymmetry). Additionally, MWMs uses an auxiliary glide to compensate joint positioning errors in active peripheral joint movements, resulting in higher-quality movement patterns (Exelby, 2002; Mulligan, 2004). According to Haavik-Taylor and Murphy (2007), manipulative therapy for the cervical spine’s periphery can influence cortical plasticity, somatosensory processing, and sensorimotor integration, which can reduce discomfort and facilitate functional reorganization. Some studies have demonstrated the efficacy of core stability training for lower back pain or SIJD (Krzyzanowicz et al., 2015; Bhat et al., 2021; Javadov et al., 2021; Moreira et al., 2021). Therefore, we infer that the MWM Techniques combined with CSE designed can improve the situation of pelvic asymmetry in patients with SIJD. Remarkably, The study protocol performed by Barbosa et al. (2013) mainly involved the correction of pelvic anteversion by quadriceps eccentric and hamstring concentric contractions. In addition, a case report from Boyle (2011) described the treatment of a female patient with chronic left low back pain and SIJ pain with a unique unilateral motor management involving activation of the hamstring or adductor muscles that corrected pelvic forward/pronation. Compared with the study of Barbosa et al. (2013) and Boyle (2011), the exercises selected in our study protocol focused more on strengthening the deep abdominal muscles and gluteus maximus muscle, and did not include specific functional exercises targeting hip joints and correcting innominate bone dysfunction. Therefore, there is small difference in pelvic tilt angle observed in SIJD participants between moments (from M0 to M1), and could explain the non-significant findings in PTAR of the current study.

### 4.1 Limitations

This study has several limitations that should be considered when interpreting the results. First, this is only a preliminary study, the sole problem of this investigation is the short duration. A longer follow-up period should be included, setting multiple moments (e.g. M2, M3, etc.). Second, the usefulness of the technique may be impacted by the therapists’ limited clinical expertise, and not all practitioners may use the MWM approach in the same way. Third, the study only used ROM kinematics data to measure lumbar ROM and movement patterns. Other measures, such as the lumbar stability index may provide additional information on lumbar mobility and the degree of spinal stability (Shamsi et al., 2017). Finally, another limitation is the small sample size, even though the number of participants are statistically sufficient, we would expand the sample size and prolong the follow-ups in the future studies.

## 5 Conclusion

This single-blind randomized controlled study showed that the CSE + MWM group participants had better outcomes in terms of pain, disability, and function. Additionally, this study offers early evidence that using MWM in a traditional low back pain regimen consisting of CSE may enhance SIJD patients’ function more effectively than CSE alone.

## Data Availability

The original contributions presented in the study are included in the article/Supplementary Material, further inquiries can be directed to the corresponding author.
