# Supplementary material for: The effects of Core Stability Exercises and Mulligan’s mobilization with movement techniques on sacroiliac joint dysfunction
Source: Front Physiol. 2024 Apr 18;15:1337754. doi: 10.3389/fphys.2024.1337754 (PMC11063399; doi:10.3389/fphys.2024.1337754)
Supplement: Supplementary file 1 [file DataSheet1.docx]

Supplementary Material

The effects of Core Stability Exercises and Mulligan’s Mobilization with Movement Techniques on Sacroiliac Joint Dysfunction

Huiqian Yan, Peng Zhao^*^, Xuanhui Guo, Xiao Zhou

*** Correspondence:** Corresponding Peng Zhao: zhaopeng@ciss.cn

# Supplementary Figures and Tables

## Supplementary Figures


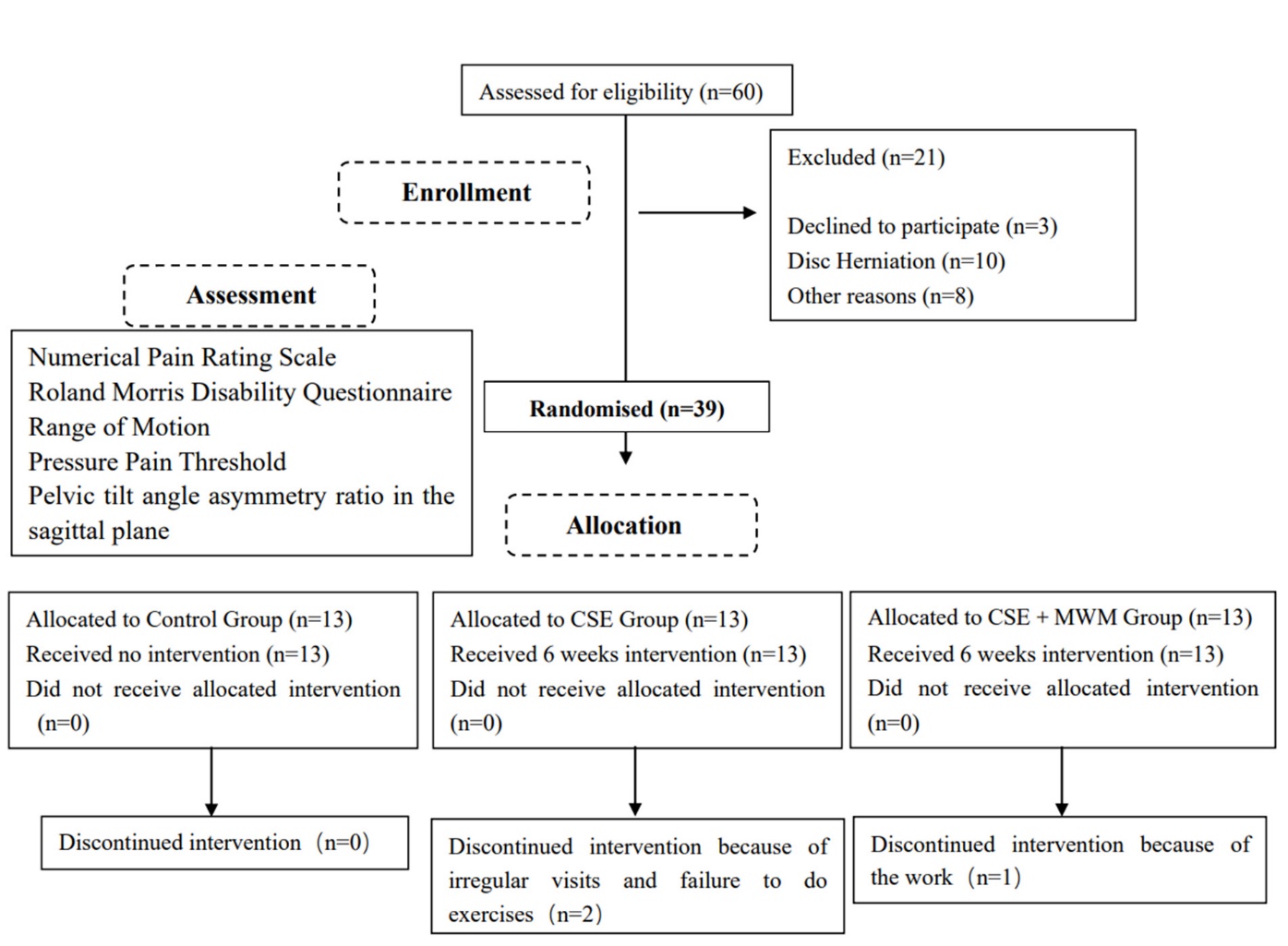


**Supplementary Figure 1.** Details of study protocol.

## Supplementary Tables

**Supplementary Table 1.** Demographic characteristics of participants.

|  |  | Control Group  （n=13） | CSE Group  （n=11） | CSE＋MWM  Group  （n=12） | Statistical test  value *F* | *P* |
| --- | --- | --- | --- | --- | --- | --- |
| Age  （years） | M±SD | 22.15±1.77 | 23.50±6.96 | 24.08±4.21 | 0.289 | 0.751 |
| Height（cm） | M±SD | 167.58±6.64 | 169.67±8.50 | 166.85±8.69 | 0.217 | 0.806 |
| Weight（kg） | M±SD | 60.15±7.77 | 59.92±7.69 | 58.65±8.44 | 0.246 | 0.783 |
| BMI（kg/m²） | M±SD | 21.00±2.08 | 20.78±1.88 | 21.00±2.00 | 0.463 | 0.634 |

BMI，Body mass index; CSE，core stability exercises; CSE＋MWM, core stability exercises with mobilization with movement; MWM, mobilization with movement; M, mean; SD, standard deviation.

*P* intergroup value: comparison between the different groups with the one-way ANOVA test.

**Supplementary Table 2.** Comparison of NPRS, RMDQ, ROM, PPT, PTAR within and between the three groups.

| Within-group |  |  |  |  |  | | Between-groups | |
| --- | --- | --- | --- | --- | --- | --- | --- | --- |
| Outcomes | Groups | M0  M±SD | M1  M±SD | M1-M0  M±SD | *P^1^* | effects | | *P^2^* |
| VAS  (mm) | CSE＋MWM | 4.25±1.14 | 0.58±0.80^a^ | -3.67±1.23 | ＜0.001 | *F*=1.424  *P*=0.255 | | 0.327  0.103 |
|  | CSE | 4.18±1.08 | 1.55±1.57^a^ | -2.64±2.25 | 0.003 |  | |  |
|  | Control | 3.38±1.85 | 1.38±2.29 | -2.00±3.37 | 0.053 |  | |  |
| RMDQ | CSE＋MWM | 3.75±2.73 | 1.50±1.83^a^ | -2.25±2.83 | 0.019 | *F*=3.153  *P*=0.056 | | 0.264  0.179 |
|  | CSE | 6.36±3.88 | 2.64±3.78^a^ | -3.73±4.08 | 0.013 |  | |  |
|  | Control | 5.62±4.09 | 5.08±3.77 | -0.54±2.33 | 0.421 |  | |  |
| Flexion  ROM  (degrees) | CSE＋MWM | 30.09±10.02 | 30.08±4.34 | -0.01±9.49 | 0.998 | *F*=0.952  *P*=0.396 | | 0.181  0.614 |
|  | CSE | 29.30±8.13 | 24.51±6.97^a^ | -4.79±6.79 | 0.041 |  | |  |
|  | Control | 33.92±9.78 | 32.20±8.60 | -1.71±8.51 | 0.480 |  | |  |
| Extension ROM  (degrees) | CSE＋MWM | 11.62±2.73 | 16.03±4.89^a^ | 4.41±6.24 | 0.032 | *F*=1.989  *P*=0.153 | | 0.355  0.055 |
|  | CSE | 14.49±5.43 | 16.12±3.82 | 1.63±7.18 | 0.467 |  | |  |
|  | Control | 15.30±6.73 | 14.05±2.38 | -1.24±7.70 | 0.571 |  | |  |
| Right lateral flexion ROM (degrees) | CSE＋MWM | 16.89±6.38 | 20.00±4.18 | 3.12±6.20 | 0.110 | *F*=1.784  *P*=0.184 | | 0.096  0.131 |
|  | CSE | 16.97±4.78 | 15.45±5.09 | -1.52±7.45 | 0.515 |  | |  |
|  | Control | 18.00±4.43 | 17.10±4.61 | -0.90±5.83 | 0.589 |  | |  |
| Left lateral  Flexion ROM  (degrees) | CSE＋MWM | 15.50±4.78 | 20.00±4.45^a^ | 4.50±4.73 | 0.007 | *F*=5.599  *P*=0.008^b^ | | 0.144  0.002^d^ |
|  | CSE | 17.00±3.89 | 17.76±6.32 | 0.76±6.75 | 0.717 |  | |  |
|  | Control | 18.41±4.06 | 14.90±4.82 | -3.51±6.34 | 0.069 |  | |  |
| Right axial  Rotation ROM  (degrees) | CSE＋MWM | 7.28±3.70 | 5.97±2.11 | -1.31±3.79 | 0.256 | *F*=0.401  *P*=0.673 | | 0.917  0.410 |
|  | CSE | 6.18±2.87 | 5.97±2.41 | -0.21±2.23 | 0.758 |  | |  |
|  | Control | 5.13±2.18 | 5.08±3.77 | -0.05±4.66 | 0.969 |  | |  |
| Left axial  Rotation ROM  (degrees) | CSE＋MWM | 6.83±3.14 | 6.61±1.92 | -0.22±2.74 | 0.788 | *F*=2.766  *P*=0.077 | | 0.029^c^  0.520 |
|  | CSE | 8.00±3.31 | 5.49±2.06^a^ | -2.52±2.38 | 0.006 |  | |  |
|  | Control | 5.31±2.15 | 4.46±1.56 | -0.85±2.09 | 0.171 |  | |  |
| PPT  (kg/cm2) | CSE＋MWM | 1.99±0.56 | 2.26±0.57 | 0.27±0.81 | 0.272 | *F*=0.589  *P*=0.560 | | 0.448  0.302 |
|  | CSE | 2.27±1.09 | 2.83±0.96 | 0.56±0.86 | 0.058 |  | |  |
|  | Control | 2.21±0.69 | 2.86±0.99^a^ | 0.65±1.00 | 0.038 |  | |  |
| PTAR  (%) | CSE＋MWM | 27.62±21.34 | 24.44±25.37 | 3.18±39.37 | 0.785 | *F*=0.686  *P*=0.511 | | 0.601  0.525 |
|  | CSE | 24.27±14.76 | 15.11±9.28 | 9.16±18.49 | 0.131 |  | |  |
|  | Control | 19.26±12.38 | 23.06±14.13 | -3.80±17.83 | 0.457 |  | |  |

*P*^1^ intragroup value: comparison between the initial moment (M0) and the final moment (M1) with the t test for paired samples; *P*^2^ intergroup value: comparison between the different groups in the difference variable (M1-M0) with the one-way ANOVA test.

CSE: core stability exercises; CSE＋MWM: core stability exercises with mobilization with movement; MWM: mobilization with movement; M: mean; NPRS: Numerical Pain Rating Scale; PPT: Pressure Pain Threshold; PTAR: pelvic tilt angle asymmetry ratio in the sagittal plane; RMDQ: Roland Morris Disability Questionnaire; ROM: Range of Motion; SD: standard deviation.

^a^Statistically significant difference within-groups, *P* < 0.05.

^b^Statistically significant difference between-groups, *P* < 0.05.

^c^Statistically significant difference between CSE＋MWM and CSE, *P* < 0.05.

^d^Statistically significant difference between CSE＋MWM and Control, *P* < 0.05.
